# Ten-Week Follow-Up of Monkeypox Case-Patient, Sweden, 2022 

**DOI:** 10.3201/eid2810.221107

**Published:** 2022-10

**Authors:** Aleksandra Pettke, Finn Filén, Katarina Widgren, Andreas Jacks, Hedvig Glans, Sofia Andreasson, Shaman Muradrasoli, Sofia Helgesson, Elenor Hauzenberger, Maria Lind Karlberg, Noura Walai, Annelie Bjerkner, Hadrien Gourlé, Sara Gredmark-Russ, Oskar Karlsson Lindsjö, Klara Sondén, Hilmir Asgeirsson

**Affiliations:** Public Health Agency of Sweden, Solna, Sweden (A. Pettke, S. Muradrasoli, S. Helgesson, E. Hauzenberger, M.L. Karlberg, N. Walai, A. Bjerkner, H. Gourlé, O. Karsson Lindsjö, K. Sondén);; Stockholm South General Hospital Skin and Venereology Clinics, Stockholm, Sweden (F. Filén);; Stockholm County Council, Stockholm (K. Widgren, A. Jacks);; Karolinska Institutet, Stockholm (K. Widgren, H. Glans, S. Gredmark-Russ, K. Sondén, H. Ásgeirsson);; Karolinska University Hospital, Stockholm (H. Glans, S. Andreasson, S. Gredmark-Russ, H. Ásgeirsson);; Laboratory for Molecular Infection Medicine Sweden, Umeå, Sweden (S. Gredmark-Russ)

**Keywords:** monkeypox, viruses, orthopoxvirus, zoonoses, epidemic, outbreak, metagenomic sequencing, Sweden

## Abstract

A previously healthy male patient had detectable monkeypox virus DNA in saliva 76 days after laboratory confirmation of infection. A comprehensive characterization of viral kinetics and a detailed follow-up indicated a declining risk for transmission during the weeks after monkeypox symptoms appeared.

Monkeypox is a zoonotic infection caused by monkeypox virus (MPXV), belonging to the *Orthopoxvirus* genus of the Poxviridae family. Monkeypox outbreaks have historically been described mainly in central and west Africa (*1*). Cases outside Africa are rare and, until 2022, consisted mostly of imported cases, patients’ household contacts, and, in some cases, nosocomial infections (*2*,*3*). One outbreak in 2003 outside Africa was linked to importing exotic pets (*4*).

In May 2022, a multinational monkeypox outbreak surfaced; cases were reported from Europe, the Americas, Israel, and Australia. Compared with those in previous outbreaks, these reported patients show a different clinical manifestation of localized rashes and mucosal lesions predominantly in the genital area. Common systemic symptoms included fever and lymphadenopathy. The cases clustered in men who have sex with men (*5*).

We report a monkeypox case detected in Sweden during the multinational outbreak, focusing on the clinical symptoms, microbial diagnostic findings, and viral kinetics in different sample types over time. Moreover, we report a fast and robust bioinformatics analysis of sequencing data for characterizing cases. We obtained consent from the patient for our study.

## The Study 

The patient, a previously healthy man with no history of smallpox vaccination, first noticed an inguinal swelling (day 0). The next day, he observed a small skin change on his foreskin, progressing over the next days to a deeper, well-circumscribed lesion with local lymphadenopathy. Fever developed on day 5 and 6, peaking at 39°C. One week after symptom onset, the patient sought care at an outpatient clinic. By then, the fever had subsided. No new lesions appeared. He reported a history of receiving oral sex from several male partners within the 3 weeks before symptom onset. At a follow-up visit on day 11, the lesion had increased in size to 2 cm in diameter. Microbiologic analyses for herpes simplex virus, syphilis, and *Haemophilus ducreyi* returned negative results; because of reports of monkeypox cases in Europe manifesting as unusual genital skin lesions, we initiated analysis for MPXV at the Public Health Agency of Sweden. We performed real-time PCRs for orthopoxvirus DNA and MPXV DNA on the genital lesion swab; results were positive and confirmed by Sanger sequencing of an orthopox-specific PCR product.

The genital lesion slowly healed but with increasing local lymphadenopathy; on day 25, the patient had a ruptured local lymph node with discharge. At a follow-up visit on day 53, the patient was feeling well but still had enlarged lymph nodes. The original genital lesion had diminished to 5 mm in diameter and bled slightly when touched. The wound from the ruptured lymph node had healed.

**Figure 1:**
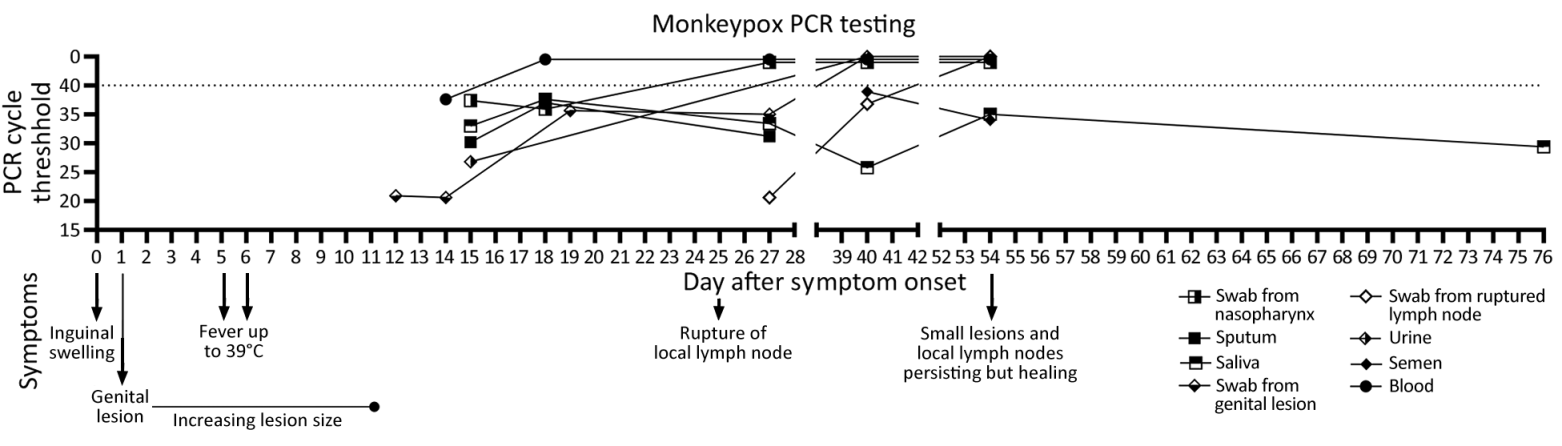
Overview of clinical and laboratory findings in a patient with monkeypox, Sweden, 2022**.** Timeline depicts clinical symptom evolution and PCR testing results. Dotted line indicates cycle threshold for detection of monkeypox virus by real-time PCR.

We took repeated samples from the patient during the 10-week follow-up period from the genital lesion, the ruptured local lymph node, urine, semen, blood and the respiratory tract. We detected MPXV DNA in most samples (Figure 1; Appendix Table 1). Although tests of all genital samples were initially positive, all showed a rapid decline in viral DNA content. Of note, MPXV DNA was detected in swabs from the ruptured lymph node 40 days after symptom onset, in semen and saliva after 54 days, and in saliva after 76 days (Figure 1; Appendix Table 1).

**Figure 2:**
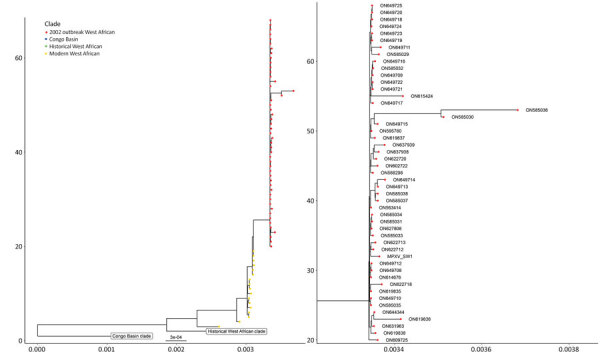
Phylogenetic tree depicting the relationship of the monkeypox virus strain detected in a genital lesion sample from a patient in Sweden to previously published isolates and the strain repsonsible for the 2022 multinational outbreak. The x-axis represents the branch lengths, interpreted as the number of nucleotide substitutions per site. The y-axis represents the tree cardinality (e.g. the amount of sequences represented in the tree) of each clade.

We performed electron microscopy on skin lesion material and observed viral particles characteristic for orthopoxviruses (Appendix Figure). The particles were 220–450-nm long and 140–260-nm wide. We extracted DNA from the first genital-lesion sample and subjected it to metagenomics sequencing using both short-read and long-read technologies. We reconstructed the viral genome from metagenomics data using a long-read first assembly approach. In brief, reads were cleaned from human sequences using Kraken 2 (https://github.com/DerrickWood/kraken2), followed by assembly of the nanopore reads using Flye (https://github.com/fenderglass/Flye), resulting in a single contig representing MPXV. The contig was polished using medaka (https://github.com/nanoporetech/medaka) for the long reads and then ntEdit (https://github.com/bcgsc/ntedit) for the short reads, which produced a nearly complete genome sequence. We compared this genome sequence by whole-genome alignment and tree construction using publicly available sequences (Appendix). The analysis suggested that the case virus belongs to the West Africa clade. Furthermore, the case is closely related with sequences reported from the current outbreak; genome alignment using ViralMSA (https://github.com/niemasd/ViralMSA) showed a single-nucleotide polymorphism distance of 4 nt (Figure 2).

## Conclusions 

As of August 2022, the multinational monkeypox outbreak is still unfolding; new cases are being reported in an increasing number of countries. Many aspects of monkeypox infection in the ongoing outbreak differ from previous endemic and imported monkeypox cases, including clinical manifestations and route of transmission (*6*,*7*). The new aspects of the infection have implications for clinical case management and behavioral recommendations for the patient, infection control measures, and public health. More knowledge is urgently needed to control the outbreak at an early stage and prevent virus transmission in previously non–monkeypox-endemic regions.

This report highlights several aspects of monkeypox as an emerging infectious disease. First, the case manifested as a single genital lesion accompanied by enlarged local lymph nodes, leading to lymph node rupture. The appearance of localized genital lesions was consistent with recent reports from other countries in Europe (*8*) and clearly demonstrated an alternative clinical manifestation of the strain of MPXV associated with the 2022 multinational outbreak, causing localized lesions rather than the classic generalized rash or vesicles spread over the body. Lymph node rupture is an unusual manifestation.

Second, we presented viral kinetics in different sample materials over time and show that, despite the localized lesion in this patient, viral DNA could also be found in urine, blood, and the respiratory tract. So far, this type of data has been published for few cases (*9*) within the current multinational outbreak, connected to sexual transmission of MPXV, but this finding is consistent with previous reports from classical monkeypox imported from Africa (*2*). The persistent detection of MPXV DNA in samples from semen and the respiratory tract in this case could have implications for transmissibility. Prolonged infectivity of bodily fluids such as semen has been described for viral infections like Zika and Ebola (*10*). However, knowledge gaps include whether a positive PCR result indicates the presence of live virus. 

Third, phylogenetic analysis revealed that the virus belongs to the Western Africa clade of monkeypox, which has been associated with lower mortality rates than the Central Africa clade (*7*,*11*). Consistent with this classification, the case-patient described had noncritical illness. Furthermore, the sequence showed high degree of similarity to recently published MPXV sequences from Portugal and other countries (*12*,*13*). 

Within the context of the emerging outbreak of monkeypox, we present comprehensive clinical and microbiologic data with long follow-up times revealing persistent PCR positivity. Previous reports have provided PCR data from single timepoints or short follow-up periods of <8 days (*9*,*14*). Moreover, we present a strategy for adequate sequencing, highlighting a fast but accurate bioinformatics analysis, combining long reads and short reads, that achieves a near-complete genome assembly (Appendix). This analysis will enable other researchers to reliably classify viruses’ phylogenetic relationships, which will lead to rapid and accurate epidemiologic case tracing and phylogenetic network analyses at a relatively low cost.

Appendix Additional information on monkeypox patient in Sweden, 2022.
