# Supplementary material for: Ten-Week Follow-Up of Monkeypox Case-Patient, Sweden, 2022
Source: Emerg Infect Dis. 2022 Oct;28(10):2074–7. doi: 10.3201/eid2810.221107 (PMC9514357; doi:10.3201/eid2810.221107)
Supplement: Appendix — Additional information on monkeypox patient in Sweden, 2022. [file 22-1107-Techapp-s1.pdf]

Article DOI: https://doi.org/10.3201/eid2810.221107

Ten-Week Follow-Up of Monkeypox Case-Patient, Sweden, 2022

Appendix

Methods

Swab and Sample Collection

Dry swabs were rolled in lesions or other sampling sites and stored at 4-8 degrees Celsius until analysis. Semen and urine were collected in sterile plastic containers without additives. Analyses were performed within 24 hours after sampling.

Total Nucleic Acid Extraction

Total nucleic acid was extracted using QIAamp DNA Blood Mini Kit (QIAGEN, https://www.qiagen.com) largely following manufacturer's instructions. A 10-minute heating step at 56 degrees was added for virus inactivation before transfer of the sample on the column. The DNA concentration of the samples was measured on a Qubit Fluorometer following the Qubit dsDNA HS Assay Kit protocol (Invitrogen, https://www.fishersci.com).

PCR Testing

Realtime PCR was performed using TaqMan Fast Advanced Master Mix (Thermo Fisher Scientific, https://www.thermofisher.com). Primers and probes are listed in Appendix Table 2. Positive and negative controls as well as inhibition and extraction controls are part of the quality control for each PCR. The method has been developed in-house at the Public Health Agency of Sweden (Solna, Sweden).

Metagenomics Sequencing

Ion Torrent sequencing was performed; briefly, Ion Xpress Plus Fragment Library Kit for AB Library Builder System (Thermo Fisher Scientific) was used to generate barcoded Ion fragment DNA libraries from totally 200 ng DNA. The libraries were amplified for 8 cycles according to manufacturer's protocol and then size-selected between 150–330 bp on a Pippin HT

using a 2% agarose gel cassettes (HTC2010, Sage Science, https://sagescience.com). The size-selected libraries were purified and quantified by an in-house real-time PCR assay (*1*). Twenty-five µl of 50 pM of libraries were used for template preparation and Ion 540 chip loading on an Ion Chef system and then sequenced on an Ion GeneStudio S5 Prime system (Thermo Fisher Scientific).

For Nanopore sequencing, 330 ng DNA was treated with RNase A (Sigma Aldrich, https://www.sigmaaldrich.com), purified using AMPure XP reagent (Beckman Coulter, https://www.beckman.com) and used for library preparation (SQK-RAD004 kit, Oxford Nanopore Technologies https://nanoporetech.com). Libraries was loaded on a R9.4.1 flow cell (Oxford Nanopore Technologies) and then sequenced using MinION MK1B and Guppy 5.1.15 in HAC basecalling mode.

Bioinformatics Recovery of Consensus Sequence from Metagenome

Sequencing data was filtered to remove human sequences using Kraken 2 version 2.1.2 using a database built on the RefSeq version of GRCh38.p13 of the human genome (*2*). Following removal of human sequences, Nanopore reads were assembled using Flye version 2.9 (running with nanopore high-quality settings) (*3*). The draft assembly was then polished using medaka version 1.6.0 (using medaka consensus with model r941_min_hac_g507) (*4*). Polished assembly was corrected for platform-specific errors using ntedit version 1.3.5 (using a 40-mer bloom filter) with the read dataset from IonTorrent (*5*).

Phylogeny

Phylogeny was built using ViralMSA for alignment toward the reference sequence (NC_003310.1), followed by iqtree using the HKY+G model, and 1,000 rapid bootstraps with bnni, reducing the risk of overestimating branch support (*6*,*7*). Additionally, cowpox (X94355.2) and horsepox (DQ792504.1) were added as outgroups for rooting the phylogeny.

The resulting consensus-tree was rerooted using gotree with cowpox and horsepox as outgroups (*8*). The following tree was pruned of the sequence KC257459.1 due to a long branch on the resulting tree.

Monkeypox outbreak genomes come from the sources listed (*9–13*), enabled by data from GenBank, INSA, and CDC.

References

1. Scaramozzino N, Ferrier-Rembert A, Favier AL, Rothlisberger C, Richard S, Crance JM, et al. Real-time PCR to identify variola virus or other human pathogenic orthopox viruses. Clin Chem. 2007;53:606–13. PubMed https://doi.org/10.1373/clinchem.2006.068635

2. Beser J, Hallström BM, Advani A, Andersson S, Östlund G, Winiecka-Krusnell J, et al. Improving the genotyping resolution of *Cryptosporidium hominis* subtype IbA10G2 using one step PCR-based amplicon sequencing. Infect Genet Evol. 2017;55:297–304. PubMed https://doi.org/10.1016/j.meegid.2017.08.035

3. Wood DE, Lu J, Langmead B. Improved metagenomic analysis with Kraken 2. Genome Biol. 2019;20:257. PubMed https://doi.org/10.1186/s13059-019-1891-0

4. Kolmogorov M, Yuan J, Lin Y, Pevzner PA. Assembly of long, error-prone reads using repeat graphs. Nat Biotechnol. 2019;37:540–6. PubMed https://doi.org/10.1038/s41587-019-0072-8

5. nanoporetech/medaka: Sequence correction provided by ONT. 2022 [cited 2022 Aug 23]. https://github.com/nanoporetech/medaka

6. Warren RL, Coombe L, Mohamadi H, Zhang J, Jaquish B, Isabel N, et al. ntEdit: scalable genome sequence polishing. Bioinformatics. 2019;35:4430–2. PubMed https://doi.org/10.1093/bioinformatics/btz400

7. Moshiri N. ViralMSA: massively scalable reference-guided multiple sequence alignment of viral genomes. Bioinformatics. 2021;37:714–6. PubMed https://doi.org/10.1093/bioinformatics/btaa743

8. Minh BQ, Schmidt HA, Chernomor O, Schrempf D, Woodhams MD, von Haeseler A, et al. IQ-TREE 2: new models and efficient methods for phylogenetic inference in the genomic era. Mol Biol Evol. 2020;37:1530–4. PubMed https://doi.org/10.1093/molbev/msaa015

9. Lemoine F, Gascuel O. Gotree/Goalign: toolkit and Go API to facilitate the development of phylogenetic workflows. NAR Genom Bioinform. 2021 Sep;3:lqab075.

10. Isidro J, Borges V, Pinto M, Ferreira R, Sobral D, Nunes A, et al. First draft genome sequence of monkeypox virus associated with the suspected multi-country outbreak, May 2022. 2022 [cited 2022 May 30]. https://virological.org/t/first-draft-genome-sequence-of-monkeypox-virus-associated-with-the-suspected-multi-country-outbreak-may-2022-confirmed-case-in-portugal/799.

11. Isidro J, Borges V, Pinto M, Ferreira R, Sobral D. Nunes A, et al. Multi-country outbreak of Monkeypox virus: genetic divergence and first signs of microevolution. 2022 [cited 2022 Aug 17]. https://virological.org/t/multi-country-outbreak-of-monkeypox-virus-genetic-divergence-and-first-signs-of-microevolution/806

12. GenBank. Draft monkeypox virus genome from confirmed monkeypox case in Massachusetts, United States, May 2022. MPXV_USA_2022_MA001. 2022. https://www.ncbi.nlm.nih.gov/nuccore/ON563414

13. GenBank. First German genome sequence of monkeypox virus associated to multi-country outbreak in May 2022. MPXV-BY-IMB25241. 2022. https://www.ncbi.nlm.nih.gov/nuccore/ON568298

Appendix Table 1. Overview of samples collected from a patient for orthopoxvirus and monkeypox virus, Sweden, 2022*

Days after symptom onset	Sample	OPXV-PCR		MPXV-PCR	
		Result	Ct value	Result	Ct value
12	Swab from genital lesion	Pos‡	21.1	Pos	20.9
14	Swab from genital lesion	Pos	22.5	Pos	20.6
	EDTA-blood†	Neg		Pos¶	38.1
15	Swab from nasopharynx	Neg§		Pos¶	38.4
	Sputum	Pos	32.3	Pos	30.2
	Urine	Pos	28.8	Pos	26.8
	Saliva	Pos	35.1	Pos	33.0
18	Serum	Neg		Neg	
	Swab from nasopharynx	Pos	39.0	Pos	36.9
	Saliva/sputum	Pos	39.2	Pos	37.0
	Saliva/sputum	Pos	39.8	Pos	37.6
19	Swab from genital lesion	Pos	38.5	Pos	35.7
27	EDTA-blood	Neg		Neg	
	Swab from nasopharynx	Neg		Neg	
	Sputum	Pos	32.1	Pos	31.2
	Saliva	Pos	34.8	Pos	33.4
	Swab from genital lesion	Pos	38.9	Pos	35.0
	Swab from ruptured lymph node	Pos	22.5	Pos	20.6
40	EDTA-blood	Neg		Neg	
	Swab from nasopharynx	Neg		Neg	
	Saliva	Pos	28.3	Pos	25.8
	Urine	Neg		Neg	
	Swab from genital lesion	Neg		Neg	
	Swab from ruptured lymph node	Neg		Pos¶	36.8
	Semen	Neg		Pos¶	38.9
54	EDTA-blood	Not analyzed		Neg	
	Saliva	Not analyzed		Pos	35.0
	Swab from ruptured lymph node	Not analyzed		Neg	
	Swab from genital lesion	Not analyzed		Neg	
	Swab from nasopharynx	Not analyzed		Neg	
	Semen	Not analyzed		Pos	34.0
76	Saliva	Not analyzed		Pos	29.4

*Detection level for both viruses by real-time PCR is 40 Ct. Ct, cycle threshold; MPXV, monkeypox virus; Neg, negative; OPXV, orthopoxvirus; Pos, positive.
†Ethylendiamintetraacetate–blood
‡Positive result.
§Negative result.
§Negative result.
¶Half the replicates tested positive.

Appendix Table 2. Primers and probes used in PCR testing of orthopoxvirus and monkeypox virus, Sweden, 2022*

Target	Sequence 5′ to 3′	Reference
OPXV_Probe	TTTTCCAACCTAAATAGAACTTCATCGTTGYGTT- FAM-TAMRA	Modified from (*1*)
OPXV_Forward	GCCAGAGATATCATAGCCGCTC	
OPXV_Reverse	ACAAAGTTTGAACAAATAGAAAAGTGTTG	
MPXV_B21R_Probe	CCGTAATCCACTTCCT-FAM-MGB	
MPXV_B21R_ Forward	GTCTACAGAGTCCAAATCCTCCTCT	
MPXV_B21R_ Reverse	TGTGGAGGAKAATAATCATCATGTT	

*MPXV, monkeypox virus; OPXV, orthopox virus.

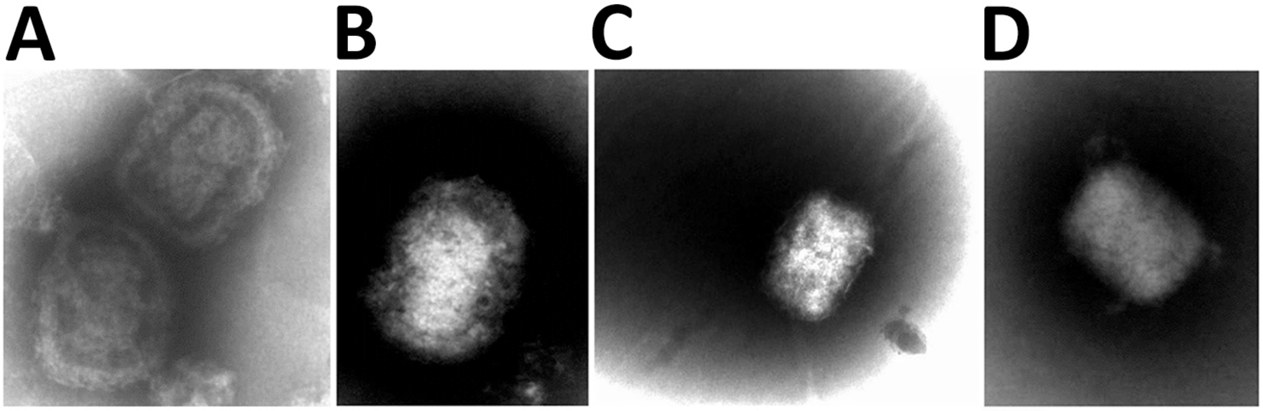

Appendix Figure. Four electron-microscope images showing orthopoxvirus-like particles found in genital-lesion samples collected 12 days after symptom onset from patient with monkeypox, Sweden, 2022.
